# Supplementary material for: Prospective Evaluation of Quality of Life and Functional Outcomes after Carbon Ion Radiotherapy for Inoperable Bone and Soft Tissue Sarcomas
Source: Cancers (Basel). 2021 May 25;13(11):2591. doi: 10.3390/cancers13112591 (PMC8199366; doi:10.3390/cancers13112591)
Supplement: Supplementary file 1 [file cancers-13-02591-s001.zip › cancers-1185659-supplementary.pdf]

Supplementary Materilas

Table S1. Late adverse events graded by CTCAE, version 4.0.

Late Adverse Events	Grade 1	Grade 2	Grade 3
Dermatitis	27	6	0
Peripheral sensory neuropathy	22	9	0
Peripheral motor neuropathy	8	2	0
Genitourinary	4	2	0
Edema	5	0	0
Gastrointestinal	2	0	0
Bone infection	0	0	1

CTCAE, Common Terminology Criteria for Adverse Events.

Table S2. MSTS and QOL from other published studies regarding BSTS.

Study	Tx. Modality	No. of Pt.	MSTS, Pre- to Post-tx.	Physical Component of Health-Related QOL, Pre- to Post-tx. (QOL Questionnaire)	Mental Component of Health-Related QOL, Pre- to Post-tx. (QOL Questionnaire)
Rivard et al. 2015 Ann. Surg. Oncol. [18]	Surgery + preoperative photon radiotherapy	52	31.4 to 33.6	40.6 to 45.1 (SF-36)	49.4 to 53.1 (SF-36)
Davis et al. 2002 JCO. [29]	Surgery + pre/postoperative photon radiotherapy	187	27.1 to 28.2	68.7 to 66.6<br>71.7 to 63.9<br>(SF-36)	70.2 to 78.3<br>62.0 to 75.8<br>(SF-36)
Davidge et al. 2009 J. Surg. Oncol [30]	Surgery	157	31.1 to 31.1	NA	NA
Leiser et al. 2016 Radiother. Oncol. [13]	Proton radiotherapy	83	NA	66.5 to 70.9 (PedQoL)	74.6 to 74.9 (PedQoL)
Coens et al. 2015 Cancer [36]	Chemotherapy	369	NA	64.1 to 57.3 (EORTC QLQ-C30)	
Hidetatsu et al. 2016 Int. J. Clin. Oncol. [10]	CIRT	7	NA to 26	NA	NA
Nishida et al. 2011 IJROBP. [26]	CIRT	10	NA to 22	NA	NA
Current study	CIRT	61	20.0 to 20.9	41.0 to 40.1 (SF-8)	43.75 to 50.3 (SF-8)

Abbreviations: MSTS = Musculoskeletal Tumor Society; QOL = Quality of life; NA = not applicable; Tx. = Treatment.
